# Covid-19 and the N95 respirator shortage: Closing the gap

**DOI:** 10.1017/ice.2020.124

**Published:** 2020-04-13

**Authors:** Daniel Nogee, Anthony J. Tomassoni

**Affiliations:** 1 Department of Emergency Medicine, Yale School of Medicine, New Haven, Connecticut

## Abstract

Due to extreme shortages of personal protective equipment caused by the COVID-19 pandemic, many healthcare workers will be forced to recycle protective masks intended for disposal after a single use. We propose investigating the use of ultraviolet germicidal irradiation to sterilize masks of SARS-CoV-2 for safer reuse.

The COVID-19 pandemic has created an unprecedented demand for disposable particulate filtering facepiece respirators (FFRs) typified by N95 respirators in widespread use. The Centers for Disease Control and Prevention has published guidelines for optimizing supply to extend stocks through limiting use, reuse at the patient and provider levels, and alternative personal protective equipment recommendations.^1^ However, these strategies may increase risk of infection in healthcare workers due to FFR contamination with SARS-CoV-2, further straining the overburdened healthcare system through the temporary or permanent loss of frontline physicians, nurses, and other healthcare professionals.

Several viral decontamination strategies have been explored to improve the safety of disposable FFR reuse without compromising protective filtration capacity and structural integrity. Testing of several variants of N95 masks included in the strategic national stockpile demonstrated that they should withstand sterilization by means of exposure to ultraviolet germicidal irradiation (UVGI), ethylene oxide, or vaporized hydrogen peroxide while maintaining appropriate protective function.^2^ UVGI has also demonstrated efficacy at significantly reducing influenza virus contamination from droplets and aerosols applied to N95 FFRs,^3^ even with mucin or sebum soiling.^4^ Although no studies have yet examined UVGI effectiveness at destroying SARS-CoV-2, it demonstrated efficacy at destroying the original SARS-CoV in viral culture media.^5^

A wide variety of UVGI facilities, including UVGI rooms and movable cabinets, are currently employed for sterilization of laboratory equipment, protective eyewear, manicure tools, microbiological materials, and more. Based on the light source employed, such devices can be calibrated via radiometry to deliver a measured amount of ultraviolet radiation per unit surface area (Joules per square centimeter) for a period sufficient to decontaminate FFRs. With appropriate instruction and oversight, smaller UVGI units may even be suitable for small facilities or point-of-care use. Single-user use of an assigned individual mask may eliminate the need to consider eradication of non–SARS-CoV-2 pathogens introduced to the mask by the user. This approach could also increase trust and confidence in the FFR reuse program because each user will know how their mask has been cared for and how many decontamination cycles it has been subjected to.

Although further work will be needed to determine dosages of UVGI to effectively sterilize SARS-CoV-2 contaminated FFRs, UVGI provides a potential avenue for greatly extending the limited FFR supply in the face of the ongoing COVID-19 pandemic in a simple, cost-effective, and rapidly deployable manner. Hospitals and healthcare facilities should consider immediate implementation of collection programs for used FFRs in anticipation of near-future sterilization and reuse programs.

## References

[1] Strategies for optimizing the supply of N95 respirators. Centers for Disease Control and Prevention website. https://www.cdc.gov/coronavirus/2019-ncov/hcp/respirators-strategy/index.html. Published 2020. Accessed March 14, 2020.

[2] Viscusi DJ, Bergman MS, Eimer BC, Shaffer RE. Evaluation of five decontamination methods for filtering facepiece respirators. Ann Occup Hyg 2009;53:815–827. 19805391 10.1093/annhyg/mep070 PMC2781738

[3] Heimbuch BK, Wallace WH, Kinney K, et al. A pandemic influenza preparedness study: use of energetic methods to decontaminate filtering facepiece respirators contaminated with H1N1 aerosols and droplets. Am J Infect Control 2011;39:e1–e9. 21145624 10.1016/j.ajic.2010.07.004

[4] Mills D, Harnish DA, Lawrence C, Sandoval-Powers M, Heimbuch BK. Ultraviolet germicidal irradiation of influenza-contaminated N95 filtering facepiece respirators. Am J Infect Control 2018;46:e49–e55. 29678452 10.1016/j.ajic.2018.02.018 PMC7115285

[5] Darnell MER, Subbarao K, Feinstone SM, Taylor DR. Inactivation of the coronavirus that induces severe acute respiratory syndrome, SARS-CoV. J Virol Methods 2004;121:85–91. 15350737 10.1016/j.jviromet.2004.06.006 PMC7112912

